# Trial‐Based Hemolysis Modeling to Investigate Operating Modes of Continuous‐Flow LVADs

**DOI:** 10.1111/aor.70021

**Published:** 2025-10-13

**Authors:** Patrick Borchers, Steffen Leonhardt, Marian Walter

**Affiliations:** ^1^ Chair for Medical Information Technology, Helmholtz Institute for Biomedical Engineering RWTH Aachen University Aachen Germany

**Keywords:** axial‐flow pump, hemolysis modeling, in vitro trials, Lagrangian approach, left ventricular assist device, model identification, power law model, speed modulation, Sputnik1

## Abstract

**Background:**

The influence of operating modes on pump‐induced hemolysis in continuous‐flow left ventricular assist devices (LVADs) can be assessed using computational fluid dynamics (CFD) simulations alongside power law models derived from shearing device experiments. However, this conventional method incurs high computational costs, limiting the exploration of diverse operating conditions and hindering online hemolysis prediction. This work presents a CFD‐free and trial‐based methodology for determining online‐capable hemolysis models for continuous‐flow LVADs.

**Methods:**

The trial‐based hemolysis model is based on a modified power law model, with parameters identified from LVAD hemolysis trials. The dynamic behavior is modeled using the Lagrangian approach. Specifically, this model was determined for the Sputnik1 LVAD and integrated with a lumped‐parameter model of the LVAD‐supported cardiovascular system. Subsequently, hemolysis was predicted across various operating modes and patient conditions.

**Results:**

The RMSE and the *R* ^2^ of the modified power law fit were 18.4 [%·mL/h] and 0.69, respectively. The relative error introduced by the Lagrangian approach was below 0.7%. For the Sputnik1, hemolysis decreased with reduced speed. Additionally, lower systemic resistance and diminished left ventricular contractility were associated with lower hemolysis, whereas speed modulation increased hemolysis across most profiles.

**Discussion:**

The proposed hemolysis model allows to assess various LVAD operating modes and patient conditions, assisting in the selection of low‐hemolysis treatment strategies. For Sputnik1 patients, it is advisable to maintain low pump speed and systemic resistance, while speed modulation should be reserved for those with low hemolysis markers. Integrating this model with online flow sensing would enable online hemolysis prediction.

## Graphical abstract

This work presents a methodology for determining online‐capable hemolysis models for continuous‐flow LVADs, derived from in vitro trials. The models are based on fitting a modified power law and applying the Lagrangian approach. They assist clinicians in selecting treatment strategies that minimize hemolysis, as demonstrated with the Sputnik1 LVAD.

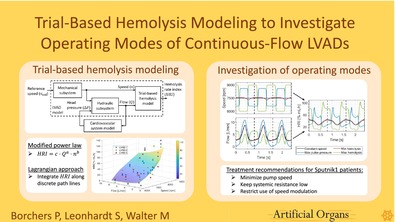

## 1 Introduction

Continuous‐flow left ventricular assist devices (LVADs) have become a standard treatment for advanced heart failure, with about 80% of implantations classified as destination therapy [1]. However, these devices induce shear stress on red blood cells (RBCs), which can lead to hemolysis [2]. Hemolysis refers to RBC damage, leading to the release of hemoglobin into plasma, which can occur either through pore formation in the RBC membrane or through complete rupture [2]. Elevated hemolysis affects 5%–18% of LVAD patients [3] and is associated with significantly reduced survival rates [4, 5]. Cowger et al. [5] reported an 8‐ to 15‐fold increase in adverse events following hemolysis detection, including pump thrombosis, stroke, right ventricular failure, and renal impairment [4, 5, 6].

Hemolysis can be quantified using the hemolysis index (HI), defined as the percentage change in plasma‐free hemoglobin (ΔpfHb) over a specified time interval, normalized by total hemoglobin (Hb) and hematocrit (HCT) [7]:

(1) $$\mathrm{HI}\;\left[\%\right]=\left(\frac{\Delta \text{pfHb}}{\mathrm{Hb}}\cdot 100\right)\cdot \left(\frac{100-\mathrm{HCT}}{100}\right)$$

The hemolysis index increases with rising shear stress (τ) and with prolonged exposure time ($t_{\exp}$), as commonly described by the power law model proposed by Giersiepen et al. [8]:

(2) $$\mathrm{HI}=c_{1}\cdot t_{\exp}^{a_{1}}\cdot \tau^{b_{1}}$$

The parameters $a_{1}$, $b_{1}$, and $c_{1}$ have been experimentally identified by various researchers using shearing devices [8, 9, 10].

Numerical predictions of LVAD‐induced hemolysis often employ computational fluid dynamics (CFD) simulations to estimate shear stresses and velocity fields. Using these results alongside the power law model from Equation (2), both Lagrangian and Eulerian approaches are established methods for quantifying hemolysis [2, 11]. The Lagrangian approach integrates the hemolysis index along discrete flow path lines, whereas the Eulerian approach integrates it across the entire flow domain with coordinates fixed in space [2, 11]. Some studies have performed CFD‐based hemolysis predictions with time‐varying flow trajectories derived from lumped parameter models of the LVAD‐supported cardiovascular system [12, 13, 14, 15, 16]. Most of these studies also explored the influence of speed modulation [13, 14, 15, 16], which is a promising strategy to enhance arterial pulsatility and reduce the risk of adverse events like bleeding and aortic valve insufficiency [17]. However, due to high computational costs, these studies focused on limited LVAD operating modes without considering varying patient conditions. Additionally, while CFD‐based predictions can illustrate relative trends, they often fall short in accurately estimating absolute values [2].

This work proposes a trial‐based and CFD‐free methodology for determining online‐capable hemolysis models for continuous‐flow LVADs. Rather than detailing the internal processes causing hemolysis, these models are intended to assess hemolysis across various LVAD operating modes and patient conditions to assist clinicians in selecting appropriate treatment strategies. The static behavior is based on a modified power law model obtained from in vitro LVAD hemolysis trials, and the dynamic behavior is modeled using the Lagrangian approach. The hemolysis model was exemplarily determined for the Sputnik1 axial‐flow LVAD [18], implanted in 49 patients by early 2020 [19]. Subsequently, the impact of various operating modes, including speed modulation, and different patient conditions on pump‐induced hemolysis was examined. The Sputnik1 axial‐flow LVAD was selected due to the availability of three specimens and prior experience in operating these devices. Moreover, due to the higher hemolysis risk of axial‐flow LVADs compared to centrifugal‐flow LVADs [3], determining operating conditions that minimize hemolysis is especially important for axial‐flow LVADs.

## 2 Methods

**FIGURE 1:**
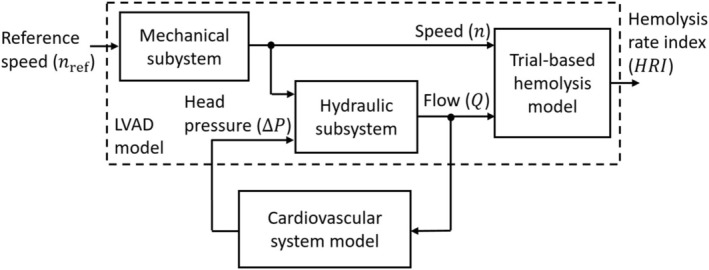
Overall model structure to evaluate the impact of LVAD operating modes on pump‐induced hemolysis.

The overall block diagram for assessing the impact of operating modes and patient conditions on LVAD‐induced hemolysis is illustrated in Figure 1. The LVAD model includes a mechanical subsystem, a hydraulic subsystem, and a trial‐based hemolysis model. The hydraulic subsystem dynamically interacts with a cardiovascular system model. All subblocks were implemented in Matlab/Simulink 2023b.

### 2.1 Mechanical and Hydraulic LVAD Subsystems

The mechanical subsystem describes the closed‐loop speed dynamics from the reference speed $n_{\mathrm{ref}}$ to the actual speed n. For the Sputnik1, these dynamics were modeled in the Laplace domain as a PT_2_ system with dead time according to Borchers et al. [20]:

(3) $$\frac{n\left(s\right)}{n_{\mathrm{ref}}\left(s\right)}=\frac{1}{3.3E\mathrm{‐06}\cdot s^{2}+0.0043\cdot s+1}\cdot e^{-0.002\cdot s}$$

The hydraulic subsystem describes the relationship between speed n, pump flow Q, and head pressure ΔP, with head pressure defined as the difference between aortic and left ventricular pressures. For the Sputnik1, it was modeled according to Telyshev et al. [21]:

(4) $$L\cdot \frac{\mathrm{dQ}}{\mathrm{dt}}=a\cdot Q+b\cdot n^{2}+c\cdot Q^{x}\cdot n^{y}-\Delta P$$

Parameters x and y were varied from −2 to 4 in steps of 1, while parameters L, a, b, and c were identified from in vitro data using linear least squares to minimize the root mean squared error (RMSE) of ΔP. The flow was estimated by integrating Equation (4) and the parameter set yielding the smallest RMSE for flow was selected. This resulted in a = −7.71 mmHg·min/L, b = 1.86E‐06 mmHg/rpm^2^, c = −5.49E‐13 mmHg·min^2^/(L^2^·rpm^3^), L = 0.74 mmHg·s·min/L, x = 2, and y = 3, with RMSE values of 0.19 L/min for Q and 3.8 mmHg for ΔP.

### 2.2 LVAD‐Supported Cardiovascular System Model

**FIGURE 2:**
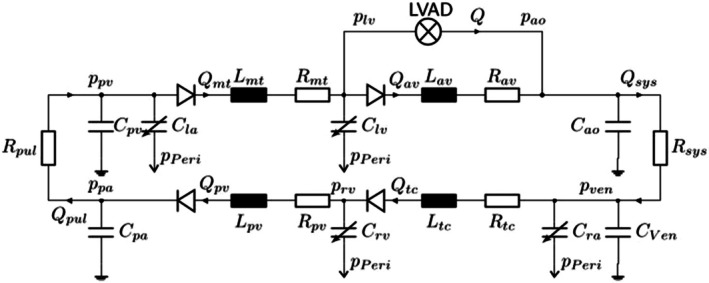
LVAD‐supported cardiovascular system model according to Heinke et al. [22]. Figure adapted from Walter et al. [23].

To investigate physiological flow conditions for LVADs, the LVAD model was integrated with a cardiovascular system (CVS) model. The selected lumped parameter model by Heinke et al. [22] consists of eight compliance compartments interconnected by resistances, inductances, and diodes that represent vessels or valves (see Figure 2). It also accounts for the interaction between the right and left ventricles through septal displacement. The model continuously calculates the aortic pressure $p_{\mathrm{ao}}$ and the left ventricular pressure $p_{\mathrm{lv}}$ based on the estimated LVAD flow Q. Cardiovascular parameters varied in this study include the contractility factor of the left ventricle ($cf_{\mathrm{lv}}$), the heart rate, and the scaling factor of the systemic resistance ($f_{\text{Rsys}}$).

### 2.3 In Vitro Hemolysis Trials

To collect data for trial‐based hemolysis modeling, in vitro testing was conducted according to the ASTM F1841 [24]. Single hemolysis trials were performed under constant conditions throughout the relevant LVAD speed and flow range. The clinically relevant speed range must be defined by the manufacturer and is usually available in the literature for established LVADs. The physiological flow range within the relevant speed range was determined using the LVAD‐supported CVS model from Chapter 2.2. For each speed, the minimum and maximum flow rates were determined across all cardiovascular parameter combinations, with $cf_{\mathrm{lv}}$ set at 0.1, 0.25, and 0.4, heart rates at 60, 90, and 120 bpm, and $f_{\text{Rsys}}$ at 0.75, 1, and 1.25.

The clinically relevant speed for the Sputnik1 ranges from 6000 to 9000 rpm [21, 25], and the determined physiological flow range is shown in Figure 4. To ensure proper blood mixing, the minimum flow rate was limited to 0.5 L/min. Hemolysis trials were conducted under 20 constant speed and flow conditions (see Figure 4), with 18 evenly distributed within the Sputnik1 operating range and two at 1 L/min for 6000 rpm and 7000 rpm to enhance model accuracy at low flow. During each trial, three Sputnik1 LVADs were operated in parallel for 6 h in automated hemolysis test benches as described by Borchers et al. [20]. Each bench was initially filled with 450 mL of porcine blood at a hematocrit of 35%. Hb, pfHb, and HCT were measured hourly as described by Borchers et al. [26].

To quantify LVAD‐induced hemolysis, the hemolysis rate index (HRI) was calculated, where ΔT is the duration between consecutive blood samples, and V is the current bench volume:

(5) $$\mathrm{HRI}\;\left[\frac{\%}{h}\cdot \mathrm{mL}\right]=\left(\frac{\Delta \text{pfHb}}{\mathrm{Hb}}\cdot 100\right)\cdot \left(\frac{V}{\Delta T}\right)\cdot \left(\frac{100-\mathrm{HCT}}{100}\right)$$

This definition, adapted from Horobin et al. [27], was normalized to the overall Hb concentration and calculated for each 1‐h interval individually. The HRI for each trial was determined as the mean value across all six intervals. The HRI represents the percentage increase in pfHb per hour, normalized to the current bench volume. Note that the Modified Index of Hemolysis (MIH), a classical hemolysis parameter obtained from in vitro studies at constant speed and pump flow (see ASTM F1841 standard [24]), can be compared to HRI values by multiplying the MIH value by the constant pump flow used in the study and a factor of 6.

### 2.4 Trials‐Based Hemolysis Model

The static behavior of the trail‐based hemolysis model is represented by a modified power law model, structurally similar to Equation (2). However, the modified model relates the hemolysis rate index HRI to the pump speed n and the pump flow Q:

(6) $$\frac{\mathrm{HRI}}{\bar{\mathrm{HRI}}}=c_{2}\cdot {\left(\frac{Q}{\bar{Q}}\right)}^{a_{2}}\cdot {\left(\frac{n}{\bar{n}}\right)}^{b_{2}}$$

The relationship between Equation (2) and Equation (6) is evident for shearing devices, where the exposure time $t_{\exp}$ solely depends on the flow rate Q, while the shear stress τ exclusively depends on the speed n [28]. The scaling parameters $\bar{n}$, $\bar{Q}$, and $\bar{\mathrm{HRI}}$ represent the mean values across all measured pump speeds, flow rates and HRI values, respectively. When combining data from multiple LVADs of the same type, the HRI values of each LVAD should be standardized by dividing through the mean HRI of the respective LVAD and then multiplying by $\bar{\mathrm{HRI}}$. The power law parameters $a_{2}$, $b_{2}$, and $c_{2}$ were identified from in vitro data using least‐squares fitting implemented via the MATLAB fit function. Bisquare weighting was applied to reduce the influence of outliers.

The Lagrangian formulation was applied to the modified power law model to incorporate dynamic properties, enabling its application to time‐varying flow and speed conditions. As a prerequisite, flow rate Q was related to exposure time $t_{\exp}$ as follows:

(7) $$Q=\frac{V_{i}}{t_{\exp}}$$

Here, $V_{i}$ denotes the blood volume between the impeller and housing, where the highest shear stresses within the LVAD are anticipated to occur [29]. Substituting Equation (7) into Equation (6) yields:

(8) $$\mathrm{HRI}={\hat{c}}_{2}\cdot {\left(t_{\exp}\right)}^{{\hat{a}}_{2}}\cdot {\left(n\right)}^{b_{2}}$$

(9) $${\hat{c}}_{2}=\bar{\mathrm{HRI}}\cdot c_{2}\cdot {\left(\frac{V_{i}}{\bar{Q}}\right)}^{a_{2}}\cdot {\left(\frac{1}{\bar{n}}\right)}^{b_{2}}$$

(10) $${\hat{a}}_{2}=-a_{2}$$

Equations ((8), (9), (10)) enable the application of the Lagrangian approach by integrating hemolysis in discrete particles moving along one‐dimensional path lines through the impeller region. At each simulation step, a new particle enters the impeller region at l=0mm, and the location l of each particle is updated according to Equations (11 and 12). Here, $l_{i}$ represents the path length of the impeller region, corresponding to the impeller length for axial‐flow LVADs. For centrifugal‐flow LVADs, $l_{i}$ could be approximated as the path length of the primary flow path as illustrated by Bourque et al. [30] (Figure 6 in [30]) for the HeartMate3 LVAD.

(11) $$l\left(t+\Delta t\right)=l\left(t\right)+\Delta l$$

(12) $$\Delta l=\frac{Q\cdot l_{i}}{V_{i}}\cdot \Delta t$$

The HRI of a particle at location l is denoted as $\mathrm{HR}I_{l}$. During each simulation step, $\mathrm{HR}I_{l}$ for each particle within the impeller region was accumulated according to Equation (13) from Goubergrits et al. [11], substituting shear rate τ for speed n. Subsequently, the effective time $t_{\mathrm{eff}}$ for each particle was calculated using Equation (14).

(13) $$\mathrm{HR}I_{l}\left(t+\Delta t\right)={\hat{c}}_{2}\cdot {\left(t_{\mathrm{eff}}+\Delta t\right)}^{{\hat{a}}_{2}}\cdot n{\left(t+\mathrm{\Delta t}\right)}^{b_{2}}$$

(14) $$t_{\mathrm{eff}}={\left(\frac{\mathrm{HR}I_{l}\left(t\right)}{{\hat{c}}_{2}\cdot n{\left(t+\mathrm{\Delta t}\right)}^{b_{2}}}\right)}^{\frac{1}{{\hat{a}}_{2}}}$$

To obtain a smooth, continuous HRI signal, the overall HRI at each simulation step was calculated by averaging the $\mathrm{HR}I_{l}$ values of all particles within the impeller region, as outlined by Equation (15):

(15) $$\mathrm{HRI}\left(t\right)=\frac{f}{N\left(t\right)}\cdot \sum_{l=0}^{l_{i}}\mathrm{HR}I_{l}\left(t\right)$$

$N\left(t\right)$ denotes the time‐dependent number of particles within the impeller region. The correction factor f accounts for deviations introduced by averaging across the impeller region. This adjustment is necessary because the integration via Equation (13 and 14) only yields identical HRI values at particle exit when compared to direct calculation via Equation (6). It was determined as the ratio between the direct and Lagrangian HRI calculations at a flow rate of 0.5 L/min.

### 2.5 Evaluating the Impact of Operating Modes

To investigate the impact of LVAD operating modes under various patient conditions, the LVAD‐supported CVS model was combined with the hemolysis model, as illustrated in Figure 1. LVAD‐induced hemolysis was evaluated for conditions without LVAD backflow and with speed profiles between 6000 and 9000 rpm. To balance model accuracy and execution time, the step time of the hemolysis model was set to Δt=0.0004s. For the Sputnik1, $V_{i}$ is 7.04 mL and $l_{i}$ is 55 mm. Furthermore, arterial pulsatility was quantified through pulse pressure, defined as the difference between maximum and minimum aortic pressure during a heart cycle. Additionally, LVAD flow and cardiac output were assessed.

The impact of the clinically established constant‐speed mode was assessed by varying the speed from 6000 to 9000 rpm in 100 rpm increments. Baseline cardiovascular conditions included a heart rate of 75 bpm, $f_{\text{Rsys}}$ of 1, and ${\mathrm{cf}}_{\mathrm{lv}}$ of 0.25. The impacts of individually varying these parameters were also evaluated.

**FIGURE 3:**
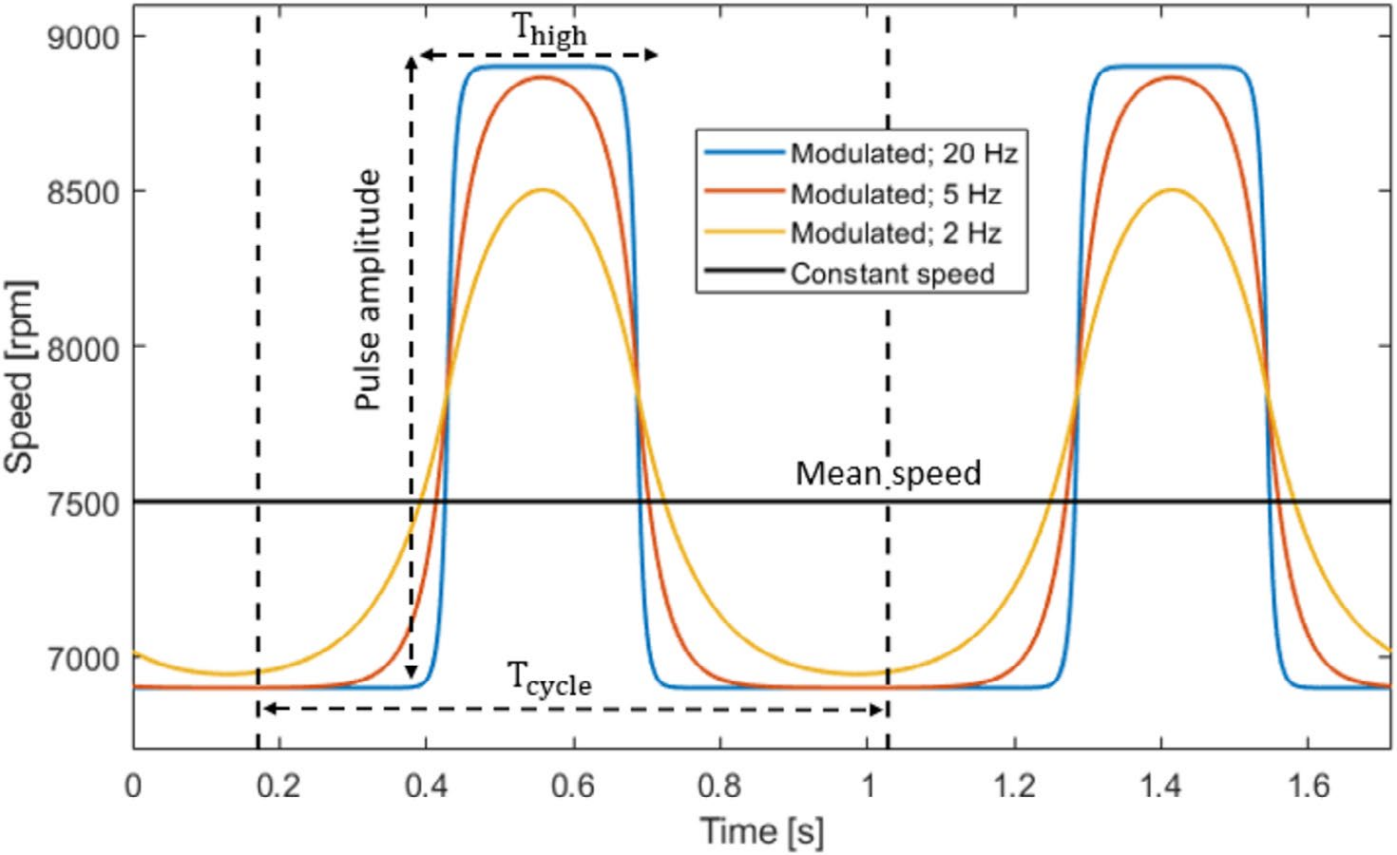
Illustration of filtered rectangular speed profiles. $T_{\text{high}}$ denotes the high‐speed duration and $T_{\text{cycle}}$ denotes the duration of the cardiac cycle. [Color figure can be viewed at wileyonlinelibrary.com]

Speed modulation was analyzed under baseline cardiovascular conditions using filtered rectangular speed profiles synchronized with the cardiac cycle (see Figure 3). The mean speed varied from 6500 rpm to 8500 rpm in 1000 rpm increments, while pulse amplitude ranged from 500 rpm to 3000 rpm in 500 rpm increments. First‐order Butterworth filters with cutoff frequencies of 20 Hz, 5 Hz, and 2 Hz were applied. The 20 Hz filter produced nearly rectangular profiles, while the 2 Hz filter yielded almost sinusoidal profiles. The ratio of high‐speed duration ($T_{\text{high}}$) to cardiac cycle duration ($T_{\text{cycle}}$) was varied in steps of 5%, and the profiles were time‐shifted by increments of 5% throughout the entire cardiac cycle.

## 3 Results

### 3.1 Trial‐Based Hemolysis Model for Sputnik1

**FIGURE 4:**
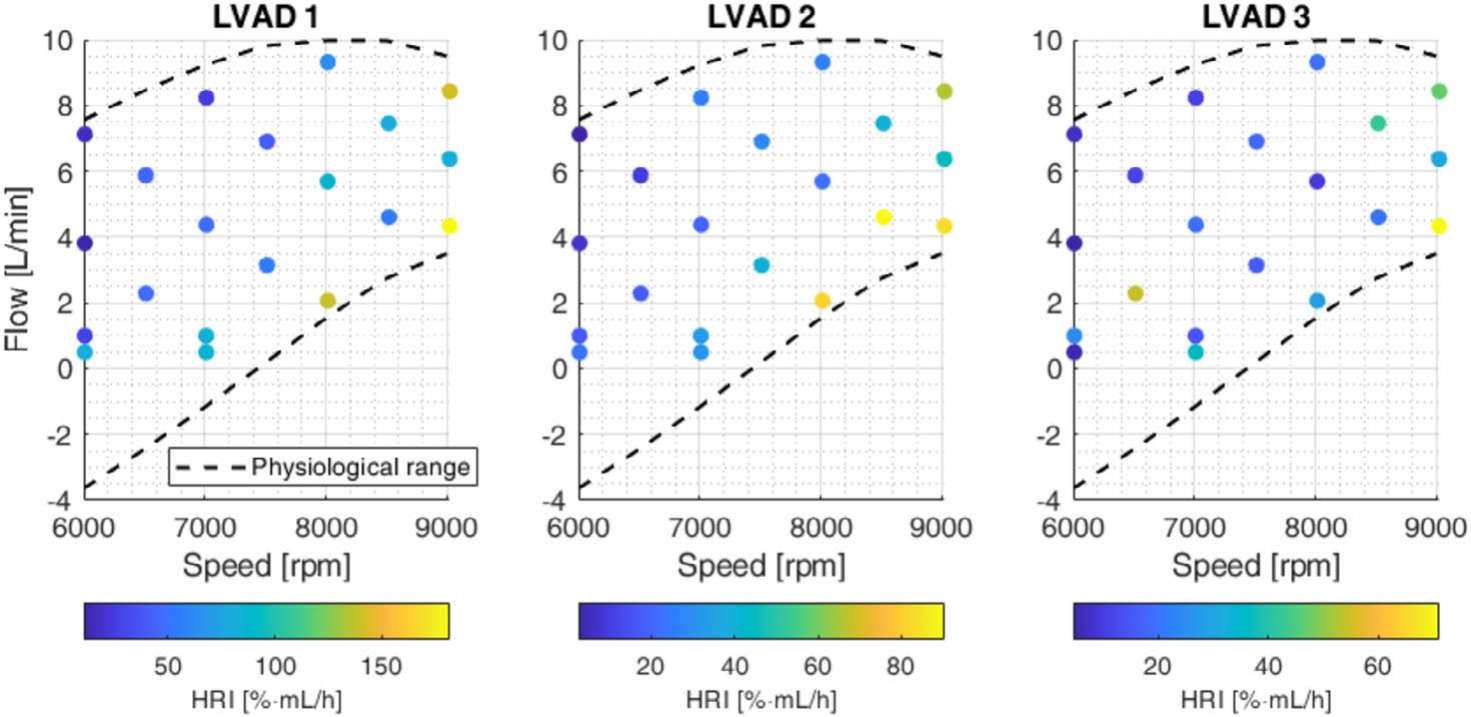
Hemolysis rate indices (HRI) obtained from in vitro hemolysis testing of three Sputnik1 LVADs across 20 constant‐speed and flow conditions. The physiological flow range under constant‐speed support is also illustrated. [Color figure can be viewed at wileyonlinelibrary.com]

The hemolysis rate indices HRI for three Sputnik1 LVADs obtained from in vitro trials are depicted in Figure 4. HRIs tend to increase with higher speeds and lower flow rates across all devices. However, the magnitudes of the HRI differed between the pumps, with LVAD 1 exhibiting higher levels than LVAD 2 and 3.

**FIGURE 5:**
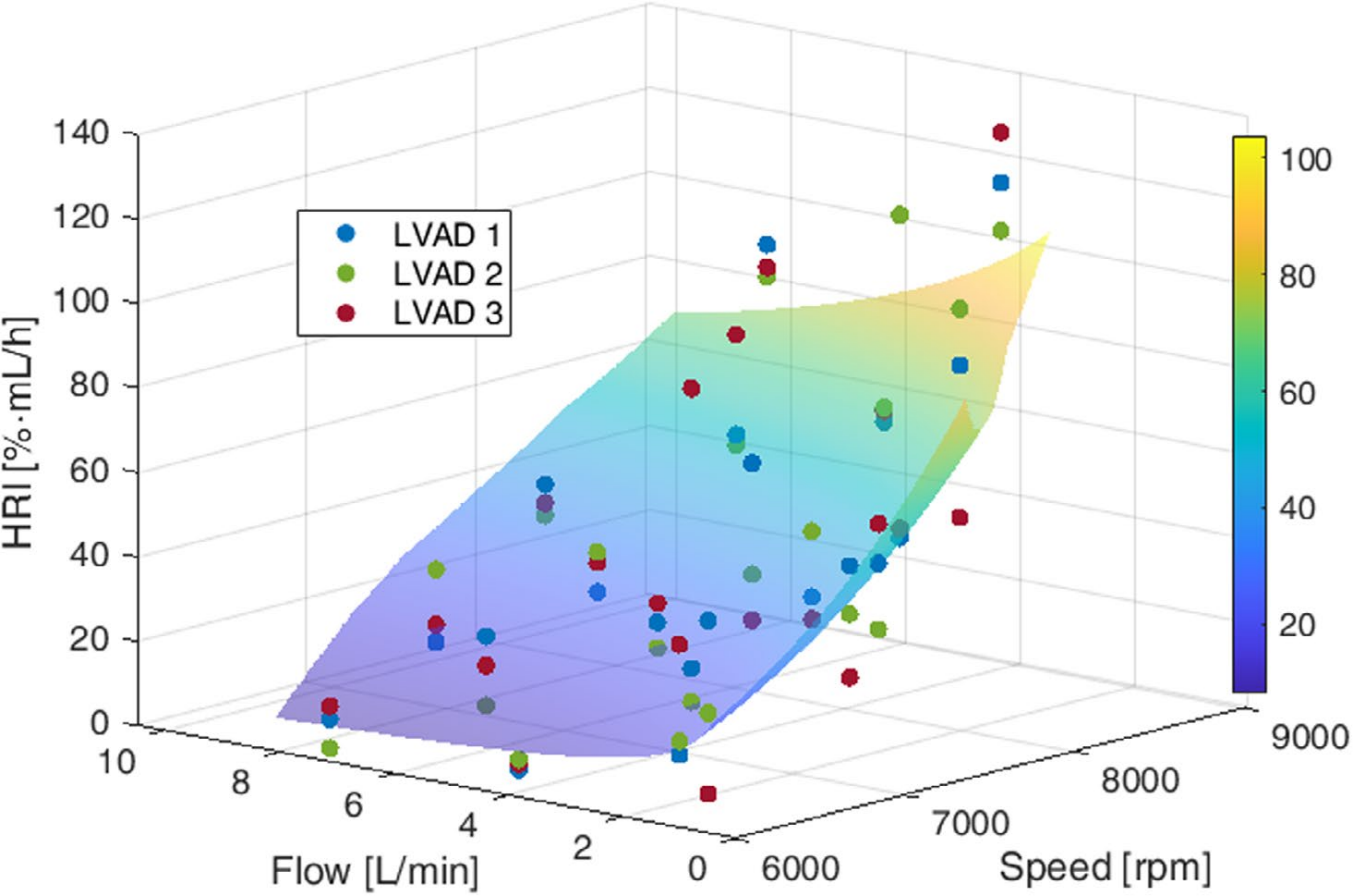
Standardized hemolysis rate indices (HRI) for all three Sputnik1 LVADs and the modified power law model. [Color figure can be viewed at wileyonlinelibrary.com]

The standardized HRI values for all three LVADs alongside the corresponding modified power law fit are shown in Figure 5. Scaling parameters and identified power law parameters are provided in Table 1. Visual inspection of the fit indicates reasonable agreement with experimental data. The RMSE and the coefficient of determination (R^2^) were 18.4 [%·mL/h] and 0.69, respectively.

**TABLE 1:** Scaling and power law parameters of the modified power law model.

	Parameter	Value	Unit
Scaling parameters	$\bar{\mathrm{HRI}}$	43.22	%·mL/min
	$\bar{Q}$	4.65	L/min
	$\bar{n}$	7415	rpm
Power law parameters	*c*_2_	0.740	—
	*a*_2_	−0.394	—
	*b*_2_	5.45	—

**FIGURE 6:**
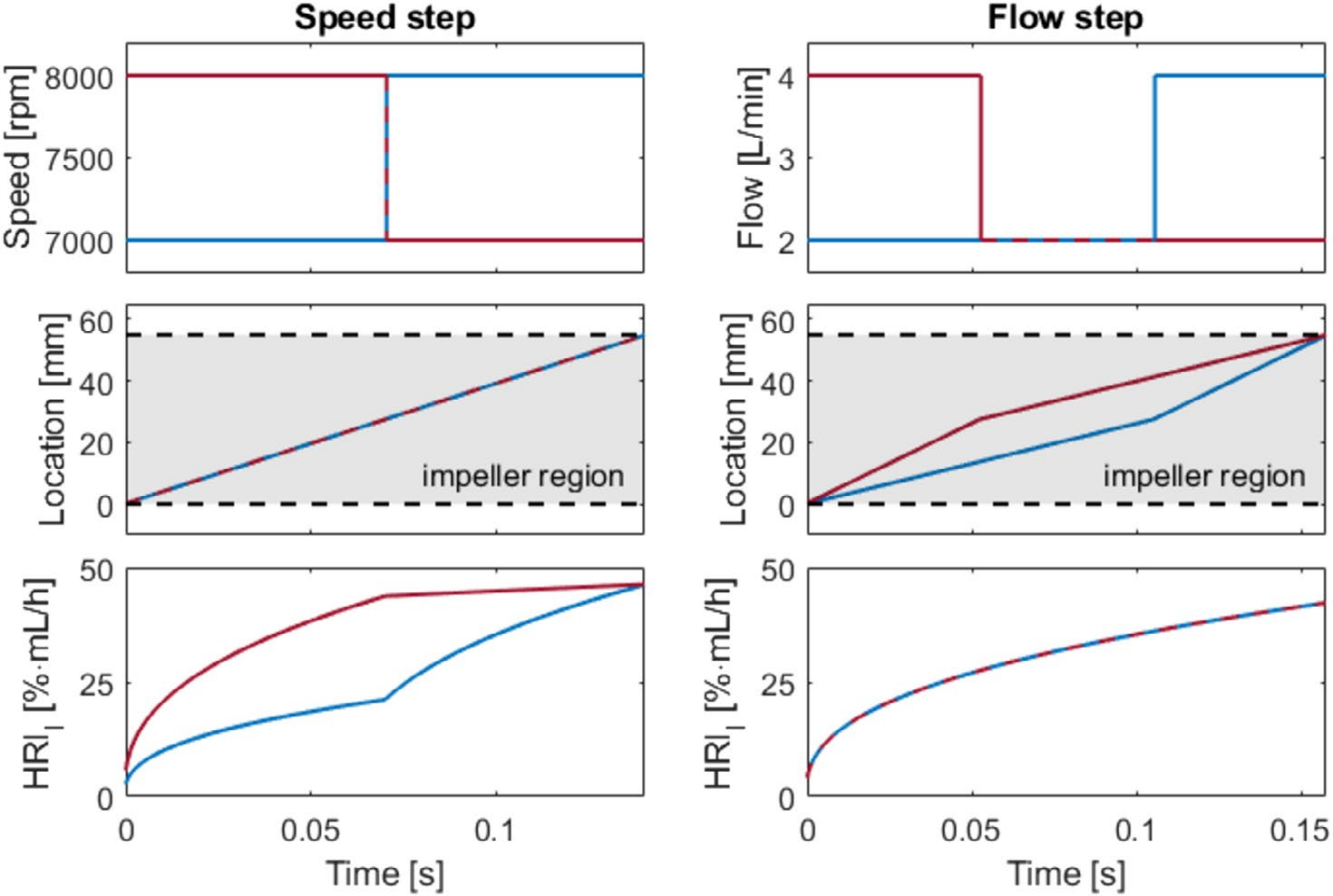
The hemolysis rate index ($\mathrm{HR}I_{l}$) and particle locations over time for different speed and flow trajectories. The left graph maintains a flow of 3 L/min, while the right graph keeps the speed at 7500 rpm. [Color figure can be viewed at wileyonlinelibrary.com]

The relative error from the Lagrangian HRI calculation remained below 0.7% for constant flow rates up to 12 L/min with a correction factor of f=1.393. Figure 6 exemplarily illustrates $\mathrm{HR}I_{l}$ and location l over time for particles moving through the impeller region (l=0mm to l=55mm) under varying speed and flow conditions. The pump speed solely affects the HRI accumulation rate, while the flow rate only influences the location change Δl. In the left graph, particles experienced a speed step, while in the right graph, they encountered a flow step at the mid‐position (l=27.5mm) of the impeller region. Notably, the particles achieve identical $\mathrm{HR}I_{l}$ values upon exiting the impeller region in both scenarios.

### 3.2 Impact of Constant Speed Level and Patient Conditions

**FIGURE 7:**
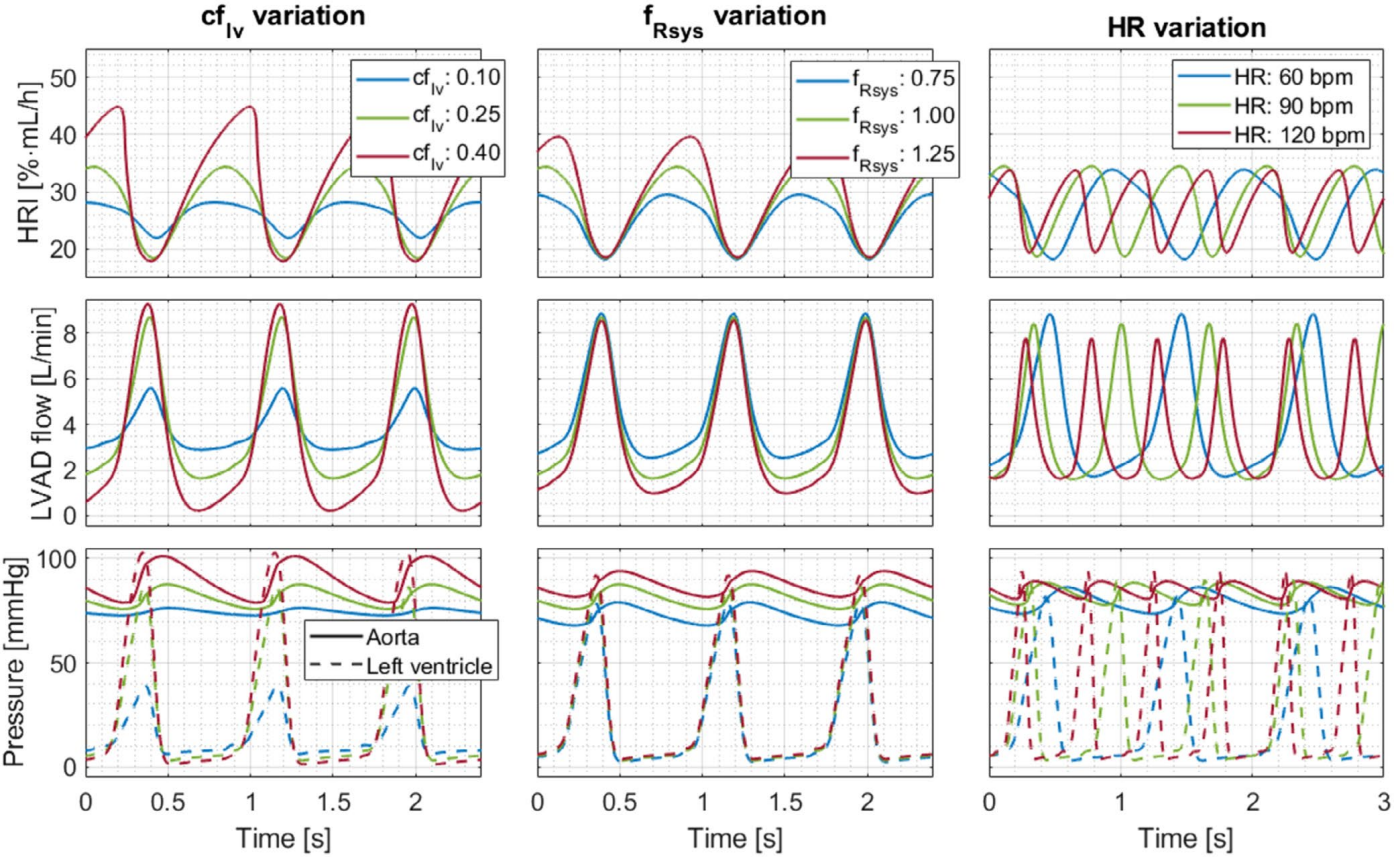
Hemolysis rate index (HRI), LVAD flow, aortic and left ventricular pressure over time at a constant speed of 7000 rpm, varying left ventricular contractility factor $\left({\mathrm{cf}}_{\mathrm{lv}}\right)$, *systemic resistance factor* $\left(f_{\text{Rsys}}\right)$ and heart rate (HR). [Color figure can be viewed at wileyonlinelibrary.com]

The upper graphs of Figure 7 illustrate the predicted hemolysis rate index HRI over time at a constant speed of 7000 rpm for various cardiovascular conditions. The middle graphs display the corresponding LVAD flows, and the lower graphs depict ventricular and aortic pressures. At a constant speed level, HRI is influenced solely by LVAD flow, which in turn depends on the cardiovascular parameters. Lower flow rates lead to longer particle exposure times, resulting in elevated HRI levels. Higher $cf_{\mathrm{lv}}$ values decreased the minimum flow rate and increased the maximum flow rate. However, comparing flow rates at $cf_{\mathrm{lv}}=0.4$ versus $cf_{\mathrm{lv}}=0.25$ reveals that the reduction in minimum flow rate is greater than the increase in maximum flow rate, leading to higher HRI values. Higher $f_{\text{Rsys}}$ values mainly decreased the minimum flow rate, also raising maximum HRI levels. Heart rate had only a minor effect on the flow range, resulting in similar HRI ranges.

**FIGURE 8:**
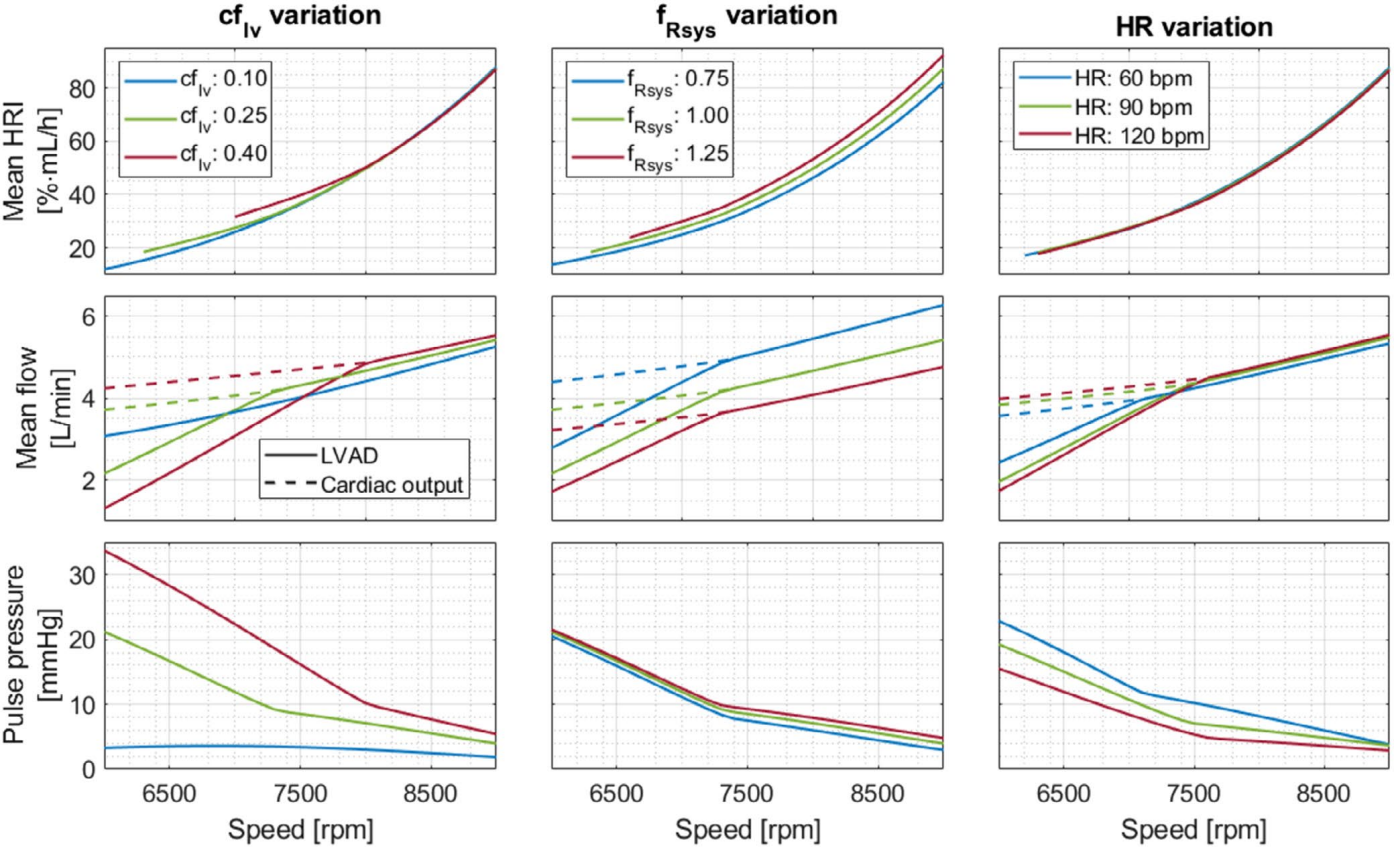
The upper graphs depict the mean hemolysis rate index (HRI) of single heart cycles at different speeds, along with varying left ventricular contractility factor $\left({\mathrm{cf}}_{\mathrm{lv}}\right)$, *systemic resistance factor* $\left(f_{\text{Rsys}}\right)$ and heart rate (HR). The middle graphs illustrate mean LVAD flow and cardiac output, while the lower graphs depict pulse pressure. [Color figure can be viewed at wileyonlinelibrary.com]

The upper graphs in Figure 8 illustrate the impact of constant speed levels on the mean HRI averaged across single heart cycles, considering variations in cardiovascular parameters. The middle graphs present the mean LVAD flow and the cardiac output, while the lower graphs depict pulse pressure. Overall, increasing speed resulted in higher mean HRI, mean LVAD flow, and cardiac output, while pulse pressure decreased across all scenarios. Higher $cf_{\mathrm{lv}}$ values increased cardiac output and pulse pressure at all speeds but reduced mean LVAD flow and increased mean HRI within the 6000 to 8000 rpm range. A higher systemic resistance factor $f_{\text{Rsys}}$ was associated with decreased LVAD flow and increased mean HRI, along with reduced cardiac output. Lastly, higher heart rates increased cardiac output but decreased LVAD flow and pulse pressure. However, heart rate had little impact on mean HRI.

### 3.3 Impact of Speed Modulation

**FIGURE 9:**
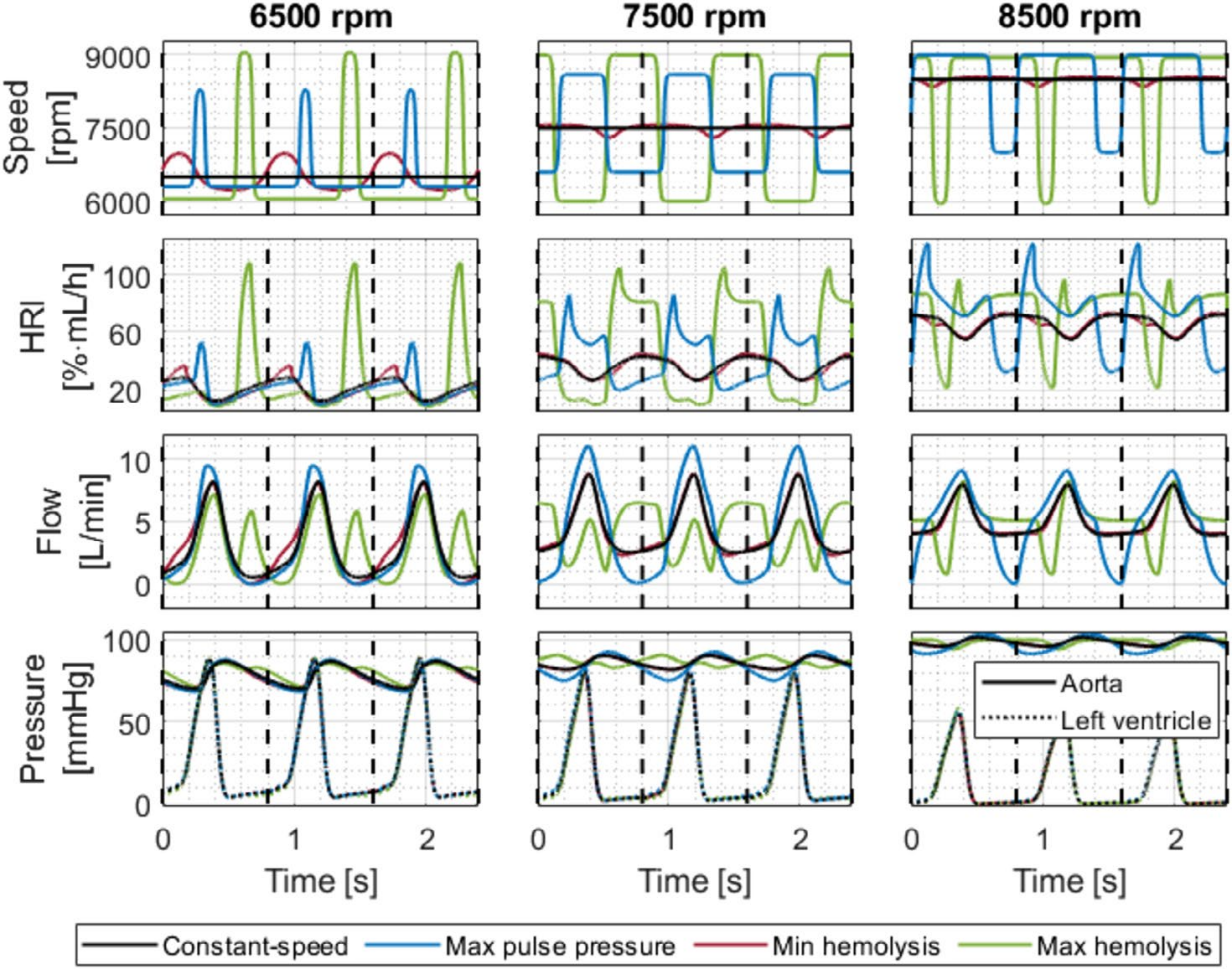
The upper graphs depict the speed profiles for constant‐speed mode, highest pulse pressure, lowest hemolysis, and highest hemolysis at mean speeds of 6500, 7500, and 8500 rpm. The graphs below illustrate the corresponding HRI, LVAD flow, left ventricular pressure, and aortic pressure over time. [Color figure can be viewed at wileyonlinelibrary.com]

Figure 9 illustrates the speed profiles for constant‐speed mode, highest pulse pressure, and both lowest and highest hemolysis at mean speeds of 6500, 7500, and 8500 rpm. It also depicts the corresponding HRI, LVAD flow, and ventricular and aortic pressures over time. The worst‐case profiles led to mean HRI increases of 27.2%, 33.0%, and 11.7% at mean speeds of 6500, 7500, and 8500 rpm, respectively. In contrast, relative decreases in HRI due to speed modulation were only 3.6%, 0.05%, and 0.005% for these speeds. The decrease at 6500 rpm is attributed to a shorter duration of low pump flow. For speed profiles maximizing pulse pressure, the mean HRI increased by 4.0%, 11.2%, and 8.8% at the same mean speeds, with corresponding pulse pressure increases of 16.5%, 106.5%, and 107.5%.

## 4 Discussion

This work presents a methodology for modeling pump‐induced hemolysis in continuous‐flow LVADs using data from LVAD hemolysis trials. While demonstrated with an axial‐flow LVAD, the universal structure of the proposed hemolysis model indicates its potential applicability to other continuous‐flow LVADs, including centrifugal‐flow pumps. However, future studies are needed for validation. Conducting studies with further LVADs would also allow for comparisons of the hemolysis impacts associated with different operating modes and patient conditions across various pumps. Such comparative analysis could play an important role in enhancing design considerations and treatment strategies, ultimately leading to a reduction in hemolysis risk for continuous‐flow LVADs. Applying the proposed methodology to other continuous‐flow LVADs requires re‐identification of the modified power law parameters and the parameters $V_{i}$ and $l_{i}$. The accuracy of CFD‐based approaches relies on precise shear stress estimation in turbulent LVAD flow, along with the selected power law parameters and the numerical integration method [2]. Conversely, the accuracy of the trial‐based method relies on test bench hemocompatibility, reproducibility of in vitro trials, and the number of trials conducted. The trial‐based methodology offers lower computational costs than CFD‐based methods, allowing for hemolysis predictions across various speed profiles and patient conditions, thereby assisting in the selection of treatment strategies that minimize hemolysis. Furthermore, by determining pump flow from sensors or internal pump parameters [31], online hemolysis prediction becomes feasible. This capability could assist clinicians in adjusting speed levels or profiles in LVAD patients with elevated hemolytic markers by providing information about the expected effect on pump‐induced hemolysis.

The trial‐based hemolysis model effectively guided treatment recommendations for Sputnik1. To minimize hemolysis in constant‐speed support, pump speed should be set as low as possible while ensuring adequate cardiac output. Moreover, systemic resistance should be kept low through adequate medication, and patients with higher residual contractility should be closely monitored for elevated hemolysis. Speed modulation increased hemolysis for most profiles, making it more suitable for patients with low hemolytic markers. This also aligns with CFD‐based studies on other continuous‐flow LVADs [13, 14, 16]. However, at low mean speeds, speed modulation may even reduce hemolysis by shortening periods of low pump flow.

## 5 Limitations

Porcine slaughterhouse blood was used as a substitute for human donor blood to avoid ethical concerns. However, porcine blood is accepted by the ASTM F1841 standard [24] and there is evidence that its behavior under device‐relevant shear stress is similar to human blood [10]. While speed modulation can increase pulse pressure, further research is needed to assess if this augmentation, which is still below healthy levels, can effectively lower the risk of adverse events. Moreover, speed modulation can lead to transiently higher flow rates than those tested in vitro, as the flow range for in vitro hemolysis testing was determined for the constant‐speed mode clinically used with Sputnik1. Flow rates below 0.5 L/min could not be experimentally assessed due to the risk of blood separation. Also backflow could not be evaluated, although it is considered undesirable as it increases RBC exposure times. Future hemolysis trials should explore time‐varying flow and speed conditions to validate the dynamic behavior of the hemolysis model introduced by the Lagrangian approach. Moreover, future research should investigate the correlation between HRI levels from in vitro studies and clinical hemolysis markers, as well as the associated risks of adverse events. To determine patient‐specific HRI values using the proposed methodology, future clinical hemolysis studies should record pump flow waveforms of LVAD patients using echocardiography [32] or estimate them from internal pump parameters [31].

## Author Contributions

Concept/design: P.B., M.W.; Data collection and statistics: P.B.; Data analysis/interpretation: P.B.; Drafting the article: P.B.; Critical revision and approval of the article: P.B., S.L., M.W.; Funding secured by: S.L., M.W.

## Conflicts of Interest

The authors declare no conflicts of interest.
